# Ovarian Cancer-Intrinsic Fatty Acid Synthase Prevents Anti-tumor Immunity by Disrupting Tumor-Infiltrating Dendritic Cells

**DOI:** 10.3389/fimmu.2018.02927

**Published:** 2018-12-14

**Authors:** Li Jiang, Xuhong Fang, Hong Wang, Diyou Li, Xipeng Wang

**Affiliations:** Department of Gynecology and Obstetrics, Xinhua Hospital, Shanghai Jiao Tong University School of Medicine, Shanghai, China

**Keywords:** ovarian cancer, FASN, tumor-infiltrating dendritic cells, immune response, immunity

## Abstract

Fatty acid synthase (FASN), the key metabolic enzyme of *de novo* lipogenesis, provides proliferative and metastatic capacity directly to cancer cells have been described. However, the impact of aberrant activation of this lipogenic enzyme on host anti-tumor immune milieu remains unknown. In this study, we depicted that elevated FASN expression presented in ovarian cancer with more advanced clinical phenotype and correlated with the immunosuppressive status, which characterized by the lower number and dysfunction of infiltrating T cells. Notably, in a mouse model, we showed that tumor cell-intrinsic FASN drove ovarian cancer (OvCa) progression by blunting anti-tumor immunity. Dendritic cells (DCs) are required to initiate and sustain T cell-dependent anti-tumor immunity. Here, our data showed that constitutive activation of FASN in ovarian cancer cell lead to abnormal lipid accumulation and subsequent inhibition of tumor-infiltrating DCs (TIDCs) capacity to support anti-tumor T cells. Mechanistically, FASN activation in ovarian cancer cell-induced the resulting increase of lipids present at high concentrations in the tumor microenvironment. Dendritic cells educated by FASN^high^ OvCa ascites are defective in their ability to present antigens and prime T cells. Accordingly, inhibiting FASN by FASN inhibitor can partly restore the immunostimulatory activity of TIDCs and extended tumor control by evoking protective anti-tumor immune responses. Therefore, our data provide a mechanism by which ovarian cancer-intrinsic FASN oncogenic pathway induce the impaired anti-tumor immune response through lipid accumulation in TIDCs and subsequently T-cells exclusion and dysfunction. These results could further indicate that targeting the FASN oncogenic pathway concomitantly enhance anti-tumor immunity, thus offering a unique approach to ovarian cancer immunotherapy.

## Introduction

Ovarian cancer (OvCa) is the leading cause of death from gynecologic cancer worldwide (1, 2). Despite advances in surgery and chemotherapy over the past years, marginally progress has been made in improving overall survival in patients with OvCa (3). Therefore, new treatment modalities and paradigms are needed to significantly improve the prognosis of women diagnosed with OvCa.

Distorted cellular metabolism has been shown to support several steps during cancer development and is emerging as a hallmark of cancer (4, 5). Recently, it has become evident that metabolic reprogramming is an important regulator of tumor sustained growth (6). In addition to aberrant glucose metabolism, *de novo* fatty acid synthesis is obviously accelerated in human malignancies. Augmented lipogenesis provides one avenue for fulfilling the demand of cancer unrestrained growth (7–9). The increased lipogenesis is represented by significantly elevated expression and hyperactivity of numerous lipogenic enzymes (7). Fatty acid synthase (FASN) is the main enzyme involved in fatty acids synthesis that catalyzes the NADPH-dependent condensation of acetyl-coenzyme A (CoA) and malonyl-CoA to produce palmitate (9). Recent evidence showed that FASN plays a crucial role in the carcinogenesis process of various cancers including OvCa (10–13). Our previous report and others recent studies have been demonstrated that fatty acid metabolism contributes to ovarian cancer tumorigenesis, which indicated a “lipid addiction” phenotype for ovarian cancers (14–16). In cancer cells, FASN confers tumor growth and survival advantages, which appears to necessarily accompany the natural history of most human cancers. FASN expression in OvCa directly promotes tumorigenesis (14, 17), however, whether it also creates a tumor-permissive immune milieu is unknown.

A growing body of research indicates that ovarian cancer shuts down the immune system which would otherwise act as the first line of defense against the deadly tumor (18–22). Understanding the link between ovarian cancer cell intrinsic events and the immune response may enable personalized immune intervention strategies for OvCa patients. Recently, large-scale analyses show that CD8^+^ TILs vary by histotype with high-grade ovarian cancers having the highest levels and a strong association with survival (20). It is well established that dendritic cells (DCs) are required to initiate and sustain T cell-dependent anti-cancer immunity. Newly, DC vaccines pulsed with autologous whole-tumor antigen has appeared as an important strategy for the mobilization of broad antitumor immunity and neoepitope-specific T cells (23). Ovarian cancer subverts the normal activity of infiltrating dendritic cells to inhibit the function of otherwise protective anti-tumor T cells (19). Re-programming or eliminating TIDCs abrogate OvCa progression (24). Several studies have also reported that metabolic reprogramming is an important regulator of the differentiation and function of dendritic cells (25). It is established that the function of dendritic cells in the tumor microenvironment is mediated by various tumor-derived factors. However, the detailed mechanism by which these factors affect DCs remains unclear. Recent several reports have revealed the importance of lipids in the function of immunosuppressive myeloid cells including dendritic cells in cancer and chronic inflammatory conditions (26–28). These data indicated that lipids could be a crucial factor in regulating the function of DCs. However, their source and the exact role of lipids in DCs of ovarian cancer activity remain unclear.

To specifically assess the effect of ovarian cell-intrinsic FASN activity in regulating the immune response, we first explore the link between ovarian cancer-intrinsic FASN expression and the accumulation of lipids in the tumor microenvironment of ovarian cancer. Moreover, we characterized the phenotype of lipid-laid DCs, and further investigated the mechanisms by which the tumor microenvironment would induce the uptake of exogenous lipids and enhance the metabolic reprogramming and dysfunctional activity of TIDCs. The results showed that upregulation of lipid accumulation in TIDCs characterized by defective profiling with impaired priming of anti-tumor T cells, which results from an increased uptake of lipids found at high concentrations in the tumor microenvironment with high FASN expression. Lipid accumulation in DCs results in inactivation of T cells, controlling a critical switch between immune stimulation and suppression. By contrast, selective inactivation of FASN partly rescues the dysfunction of dendritic cells induced by lipid accumulation.

## Materials and Methods

### Animal Model

Female C57BL/6 mice (6–8 weeks old) and athymic C57 nude mice (6–8 weeks old) were purchased from Shanghai Laboratory Animal Center of China (Shanghai, China). All mice were maintained in a pathogen-free animal facility for at least 1 week before each experiment. For the ID8 model, 2 × 10^6^ cells were used subcutaneously in C57BL/6 mice or nude mice. Cerulenin was obtained from Sigma (USA). *In vivo* experiments, treatment with cerulenin at 30 mg/kg was given i.p. at days 1, 4, and 7 after tumor inoculation in the cerulenin group. For the later treatment group, cerulenin was given i.p. at days 7, 10, and 13 at 30 mg/kg after tumor inoculation. For a collection of ascites, parental or different FASN expressed ID8 intraperitoneal ovarian tumors were generated as previously described (18). Briefly, 2 × 10^6^ tumor cells were injected into wild-type C57BL/6 mice. Implanted animals progressively developed multiple peritoneal masses and eventually massive ascites in 1.5–2 months. Mice were weighed weekly to monitor malignant ascites accumulation and animals with severe abdominal distension were humanely euthanized. All animal experiments were undertaken with review and approval from the Animal Ethical and Experimental Committee of Xinhua Hospital, Shanghai Jiao Tong University School of Medicine.

### Clinical Samples and Database

Fresh ovarian tumor tissues, ascites, and autologous peripheral blood were obtained from 50 patients with ovarian cancer who underwent surgical resection at Xinhua Hospital, Shanghai Jiao Tong University School of Medicine, China. Ascites from stage III or IV epithelial ovarian cancer patients was obtained either during debulking surgery or via ascites drainage. None of these patients had received chemotherapy or radiotherapy before surgery. All patients were diagnosed by pathological analyses based on the International Union against Cancer (UICC)-defined TNM criteria. The study protocol conformed to the ethical guidelines of the Declaration of Helsinki and was approved by the Institutional Review Board and Ethics Committee of Xinhua Hospital, Shanghai Jiao Tong University School of Medicine, China. In addition, we performed a bioinformatics analysis on the basis of microarray data retrieved from Oncomine online databases (29, 30) using a primary filter for ovarian cancer, sample filter to use clinical specimens and dataset filters to use mRNA datasets with more than 368 patients. Patients of all ages, gender, disease stages or treatments were included. We also used tumor immune estimation resource (TIMER) (31) to comprehensively investigate the molecular characterization of tumor-immune interactions in ovarian cancer.

### Cell Culture

The mouse ovarian surface epithelial cell line ID8 was obtained from Millipore Sigma (SCC145, MA, USA) and cultured in DMEM:F12 (Gibco, Life Technologies, USA) containing 10% FBS (Gibco, USA), 100 U/ml penicillin, and 100 μg/ml streptomycin at 37°C in a humidified atmosphere of 5% CO_2_. All cultured cells were tested and found to be negative for mycoplasma contamination.

### Immunohistochemistry

Standard immunohistochemically staining on human samples were performed using biopsies from ovarian cancer patients by the VECTASTAIN Elite ABC system (Vector Laboratories, Burlingame, CA, USA) according to the manufacturer's protocol. Anti-FASN polyclonal antibody (Abcam, USA), CD8-specific monoclonal antibody (CD8, Dako) was used as primary antibody. the number of CD8-positive T cells within samples (2.5 mm diameter) was counted using ImageJ cell counter and calculated as a number of CD8^+^ T cells per mm^2^. Samples with fewer than 50 CD8^+^ T cells per mm^2^ were considered T-cell-infiltrate low, whereas counts >50 per mm^2^ were considered as T-cell high. All slides were scored by two observers blinded to the pathology and the clinical features.

### The Generation of BMDCs and TIDCs

Murine bone marrow-derived dendritic cells (BMDCs) were generated from C57BL/6 mice bone marrow and cultured for 7 days with 20 ng/ml GM-CSF and IL-4 (R&D Systems, USA). Media was changed every 3 days. Mouse ID8 cells (5 × 10^5^) were injected into the abdominal cavities of C57BL/6 mice in 100 μl of DMEM and allowed to grow 10–14 days. Tumor tissue was collected for tumor-infiltrated dendritic cells (TIDCs) separation. In some experiments, enrichment of dendritic cells from the peritoneal cavity of controls or tumor-bearing mice were incubated with 10 μl magnetic microbeads conjugated to an antibody against CD11c (Miltenyi) per 10^7^ cells for 15 min at 4°C. Cells with magnetic beads were then removed from the cells suspension.

### Tumor Supernatant Preparation and Collection

Tumor explants were prepared from freshly isolated subcutaneous tumors or peritoneal transplant tumor. ID8 tumor explants were removed after euthanizing the mice. Tumors were digested in 1 mg/ml collagenase Type IV (Roche), 0.25% DNase Type I (Roche) and 1% hyaluronidase at 37°C for 1 h. Tumor samples were then pressed through a 70 μm nylon filter (BD Biosciences) to create a single cell suspension. Cells were cultured in RPMI 1640 with 10% FBS and 1% penicillin plus streptomycin overnight. The cell-free supernatant was collected to prepare tumor explant supernatant (TES). ID8 cells were grown in a DMEM-complete medium. After 1 day, the medium was recovered and filtered through a sterile 0.22 μm syringe filter to prepare tumor-conditioned medium (TCM).

### Lipid Staining

The sorted TIDCs or *in vitro* treated dendritic cells were cytospinned onto glass slides at 1,000 rpm, for 5 min. Slides were dried for 10 min, and then immediately were fixed with 4% paraformaldehyde (PFA). For lipid staining, the slides were stained with BODIPY 493/503 (ThermoFisher Scientific) at 0.5 μg/ml for 15 min at RT. Finally, slides were washed with PBS before nuclear staining with DAPI (Life Technologies) for 2 min, washed in PBS, dried, and then imaged with immunofluorescence microscopy.

### Lipid Profiling Assay

For lipidomic analysis, supernatants from parental, FASN^low^ (shFASN), or FASN^high^ (shCtrl) ID8 malignant ascites or normal peritoneal fluid was harvested, and fatty acids and triacylglycerols were extracted and quantitatively analyzed via LC-MS at the Lipidomics Core Facility of Shanghai Jiao Tong University.

### Isolation of Tumor-Infiltrating Cells

Mouse tumor samples were minced with scissors before incubation with 1 mg/ml collagenase (Roche) and 50 U/ml DNase I (Roche) in RPMI for 30 min at 37°C with agitation. Tumor samples were homogenized by repeated pipetting and filtered through a 70 μm nylon filter (BD Biosciences) in RPMI supplemented with 10% FCS to generate single-cell suspensions. Cell suspensions were washed once with complete RPMI and purified on a Ficoll gradient to eliminate dead cells. Cells from mouse spleens were isolated by grinding spleens through 40-μm filters. After red blood cell (RBC) lysis, all samples were washed and re-suspended in FACS buffer (PBS/0.5% BSA) or RPMI depending on further use.

### Flow Cytometry Assay

Immune cells isolated from mouse tumors, spleens, ascites or *in vitro* cultured condition were pre-incubated with the anti-CD16/32 monoclonal antibody (Fc block, BD Biosciences) to block non-specific binding and then stained with appropriate dilutions of various combinations of fluorescently labeled antibodies for 30 min on ice. All experiments with BODIPY 493/503 staining were performed as previously described (32). For all fluorescence channels, positive, and negative cells were gated on the basis of Fluorescence minus One controls (FMOs). All flow experiments were performed on FACS Canto II machines (BD). All antibodies used for flow cytometry are listed (Supplementary Table S1). All analysis was performed using FlowJo version 10 (FlowJo LLC).

### Assessment of Dendritic Cell Antigen Uptake by Flow Cytometry

The endocytic activity of *in-vitro* generated BMDCs or TIDCs was assessed measuring the uptake of the fluorescent reporters DQ-OVA (ThermoFisher Scientific, USA) as previously described (33). DQ-OVA is a mannose receptor ligand consisting of naturally mannosylated OVA extensively labeled with the fluorochrome. Fluorescent detection will occur if DQ-OVA degrades by DCs. Briefly, 2 × 10^5^ DC cells/ml were suspended in 100 μl complete conditioned medium and incubated with 25 μg/ml DQ-OVA at 37°C or at 0°C for 15 min. The incubations were stopped by adding 2 ml cold FACS buffer. The cells were washed twice with FACS buffer, and their fluorescence was analyzed using flow cytometry.

### T Cells Stimulation

Mouse tumor single-cell suspensions were generated as described in the previous section. Cells were stained with anti-CD45-Alexa-Fluor-780 and anti-CD3-APC for flow cytometry sorting for CD3^+^ T cells (CD45^+^CD3^+^) on FACSAria II Cell Sorter (BD). Dead cells were excluded using DAPI. The purity of flow-sorted populations was above 95%. 2.5 × 10^5^ sorted CD3^+^ T cells from the spleen of C57BL/6 mouse were stimulated on plates coated with 2 μg/ml anti-CD3 antibody (Biolegend) and soluble 1 μg/ml anti-CD28 antibody (Biolegend) in the T-cell medium for 8 h. Following indicated incubation, cells were harvested and suspended in TriZol Reagent (Invitrogen) for subsequent RNA isolation, and supernatants were stored at −80°C immediately and used for cytokine assay. For sorting CD8^+^ naïve T cells, spleen cells of B6 mouse were stained with anti-CD45A-Alexa-Fluor-780, anti-CD3-APC, anti-CD8-PE antibodies for flow cytometry and CD45A^+^CD3^+^CD8^+^ cell were sorted on FACSAria II Cell Sorter (BD). Dead cells were excluded using DAPI. The purity of flow-sorted populations was above 95%.

### T Cells Suppression Assay

CD8^+^ T cells were purified as described in the previous section and then labeled with 5 μM CFSE (Biolegend) in PBS for 6 min at 37°C. The CFSE-labeled CD8^+^ T cells were then plated in complete RPMI media onto round bottom 96-well plates (2.5 × 10^4^ cells per well) coated with 2 μg/ml anti-CD3 and 1 μg/ml anti-CD28 antibodies (Biolegend). Purified dendritic cells were added in indicated ratios (1:10) and plates were incubated at 37°C. The CD8^+^ T cell proliferation is determined by Cell Trace Violet dye dilution measured by flow cytometry (FACS Canto II, BD) after 72 h.

### Cytokine Quantification

Mouse IL-2 (R&D) and mouse IFN-r (R&D) were quantified from T cell culture supernatants using enzyme-linked immunosorbent assays (ELISA) following manufacturer's protocols (R&D Systems, USA). All ELISAs were done using 96-well high-binding Microlon 600 ELISA plates with a lower limit of quantification of 16.5 pg/mL (Greiner Bio-One, NC, USA) and plates were read using a Synergy HTTR microplate reader (Bio-Tek, USA).

### Gene Expression

Fluorescent-activated cell sorting (FACS) was conducted on a FACS Aria-II (BD Biosciences), with 100,000 sorted cells flash frozen in liquid nitrogen as a cell pellet. For real-time PCR analysis RNA was prepared using RNA Isolation Kit (Invitrogen, USA). The quality and concentration of RNA were assessed with Nanodrop Spectrophotometer (Thermo Fisher Scientific, USA). RNA reverse transcription used Prime-Script RT master mix (Takara, Japan). The RT-PCR analysis of indicated genes was performed using 7900 HT Real-Time PCR with SYBR Premix Ex Taq (Takara, Japan) in triplicate.

### Statistical Analyses

Statistical analyses were performed with a Mann–Whitney test when comparing the means of two independent groups and two-way ANOVA when comparing more than two groups. *P* < 0.05 was considered statistically significant (^*^P < 0.05, ^**^P < 0.01, ^***^P < 0.001). All statistical analyses were done with GraphPad Prism 7.0 software.

## Results

### FASN Was Upregulated in Human Ovarian Cancer and Negatively Correlated With Anti-tumor T Cell Infiltration

**Figure 1:**
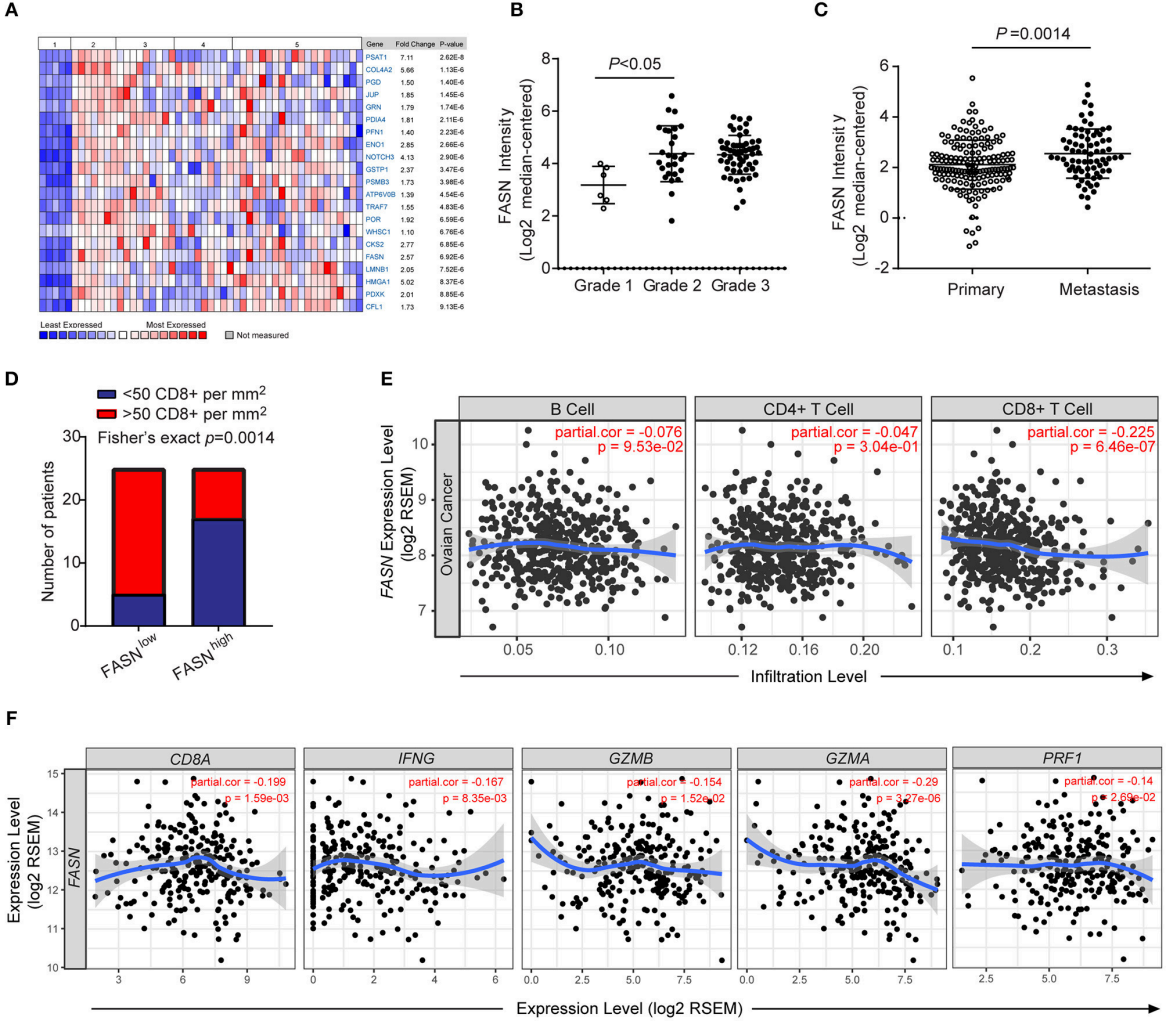
FASN was upregulated in human ovarian cancer and negatively correlated anti-tumor functional T cell infiltration. **(A)** Heatmap depicted top genes list increased in different types of human ovarian cancer compared to the normal ovarian surface epithelium. 1. Ovarian Surface Epithelium (*n* = 5); 2. Ovarian Clear Cell Adenocarcinoma (*n* = 7); 3. Ovarian Endometrioid Adenocarcinoma (*n* = 9); 4. Ovarian Mucinous Adenocarcinoma (*n* = 9); 5. Ovarian Serous Adenocarcinoma (*n* = 20). **(B)** FASN expression was upregulated in a higher grade of human ovarian cancer. (Grade 1, *n* = 6; grade 2, *n* = 27; grade 3, *n* = 58). **(C)** The expression of FASN was increased in OvCa patients with metastasis (Primary patients, *n* = 166, metastatic patients, *n* = 66). **(D)** Correlation between the expression intensity of FASN and distribution of CD8^+^ T cells in human ovarian cancer biopsies. Fisher's exact test with *n* = 50 (FASN low, *n* = 25; FASN high, *n* = 25). **(E)** The correlation of FASN mRNA expression with tumor-infiltrating B cells, CD4^+^ T cells and CD8^+^ T cells in human ovarian cancer based on TIMER analysis from TCGA datasets. The scatterplots showed the purity-corrected partial Spearman's correlation and statistical significance. **(F)** The correlation of FASN mRNA expression with gene signature of CD8^+^ T cells (*CD8A, IFNG, GZMB, GZMA*, and *PRF1*) in human ovarian cancer based on TIMER analysis. The scatterplots showed the purity-corrected partial Spearman's correlation and statistical significance.

Oncomine data-mining analysis revealed that upregulation of FASN was presented in 9 of 20 cancer types, especially in ovarian cancer, bladder cancer, colorectal cancer, and prostate cancer (Supplementary Figures S1A,B). Consistent with our previous study (14), FASN was one of the top increased genes in human ovarian cancer (Figure 1A). Using Meyniel's dataset, we showed that the mRNA level of FASN was significantly increased in higher grade patients with ovarian cancer (Figure 1B). Similarly, the bioinformatics analysis from 308 OvCa patients showed that FASN mRNA levels were significantly upregulated in the patients with metastasis compared to non-metastatic patients (Figure 1C).

To explore the effect of FASN upregulation on tumor microenvironment (TME), we analyzed the expression of FASN in human ovarian cancer tissues by immunohistochemistry (IHC). We observed an inverse association between FASN expression in tumor cells and the infiltration of CD8^+^ cytotoxic T cells (CTLs) (Figure 1D). We furtherly utilized the resource of Tumor-Immune Estimation Resource (TIMER) (31) to comprehensively investigate molecular characterization of tumor-immune interactions. TIMER analysis clearly showed a significant negative correlation between FASN gene and CD8^+^ T cells in OvCa (Figure 1E). Immune signature genes have also been used to characterize immune infiltrates (34). We measured effector cell cytolytic activity using the transcript levels of five genes (CD8A, IFNG, GZMB, GZMA, and PRF1) to elucidate the association of FASN expression and immune evasion. Additional analysis revealed a significant negative correlation between FASN gene and CD8A transcripts, as well as related cytotoxic genes which were functional transcripts of the CD8^+^ cytotoxic cells (Figure 1F). Bioinformatics analysis also revealed a negative correlation between FASN gene and dendritic cells transcripts (Supplementary Figure S1C). A similar observation was made in human prostate and bladder cancer which presented higher FASN expression (Supplementary Figure S1D), confirming its wide application. In sum, these data indicated an inverse correlation between ovarian cancer-intrinsic FASN level and tumor infiltration by CD8^+^ CTLs in human ovarian cancer.

### OvCa-Bearing Mice With Elevated FASN Expression Showed T Cell Exclusion and Defective Tumor-Infiltrating DCs

**Figure 2:**
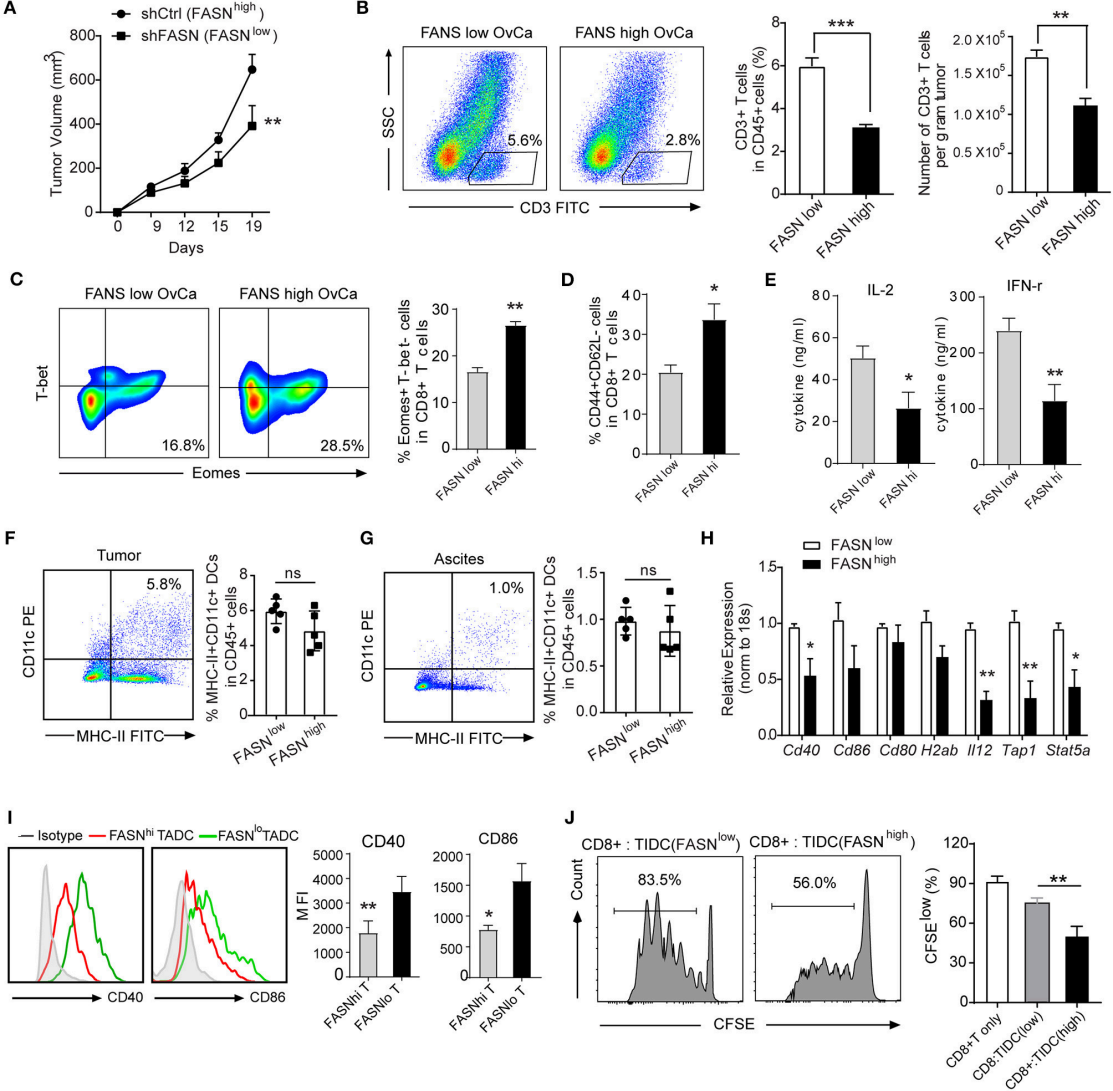
Ovarian cancer with elevated FASN expression showed T cell exclusion and defective tumor-infiltrating DCs. **(A)** Tumor growth in synergetic mice after the inoculation of ID8 cell transfected with vehicle control (shCtrl: FASN^high^) or knocking-down FASN (shFASN: FASN^low^). **(B)** Representative example out of five for flow cytometry analyzing tumor-infiltrating CD3^+^ T cells in ID8 tumor-bearing mice with high (FASN^high^) or low expression of FASN (FASN^low^) on day 15. Statistical bar graph showed CD3^+^ T cells depicted as percentage living CD45^+^ cells and absolute numbers per gram tumor. *n* = 10. **(C)** Representative color contour plots examining Eomes^+^T-bet^−^ exhausted CD8^+^ T cells from mice with shCtrl-ID8 or shFASN-ID8 tumor, and quantification of Eomes^+^T-bet^−^ cells in CD8^+^ T cells was shown. **(D)** Quantification of tumor infiltrated CD44^+^CD62L^−^ cell population in CD8^+^ T cell from tumor-bearing mice transplanted with shFASN-ID8 or shCtrl-ID8 (*n* = 10). **(E)** Analysis of cytokine IL-2 and IFN-r in the supernatant of sorted CD3^+^ T cells isolated from FASN^low^ or FASN^high^-ID8 tumors with anti-CD3/CD28 stimulation (*n* = 10). **(F,G)** Flow cytometry analysis and quantification of MHC-II^+^CD11c^+^ dendritic cells in the tumor **(F)** and ascites **(G)** of mice with the inoculation of shCtrl-ID8 (FASN^low^) or shFASN-ID8 (FASN^high^) (*n* = 10). **(H)** mRNA expression of indicated DCs associated genes in TIDCs sorted from ID-8 transplanted mice with FASN^low^ or FASN^high^. The results were normalized to the level of expression of 18sRNA. **(I)** Representative flow cytometry analysis and quantification of CD40 and CD86 on MHC-II^+^CD11c^+^ cells from tumor-bearing mice transplanted with FASN^low^ ID8 or FASN^high^ ID8 cells. (*n* = 10). **(J)** *In vitro* immunostimulatory activity of tumor-infiltrating CD11c^+^ cells purified from FASN^low^ or FASN^high^ ID-8 tumor-bearing mice. Representative histograms of CD8^+^ T cell proliferation at CD8^+^ to CD11c^+^ cell ratio 10:1 (left panel) and quantification of CD8^+^ T cell proliferation using CFSE dilution (right panel) (*n* = 3). Data presented as mean ± SEM; representative of at least 3 independent experiments; ns, no significance; ^*^P < 0.05; ^**^P < 0.01; ^***^P < 0.001.

To investigate directly whether FASN signaling within tumor cells could adversely affect anti-tumor T-cell responses, we use an immunocompetent syngeneic mouse model transplanted mouse ovarian cancer cell ID8 with or without knockdown FASN (Supplementary Figure S2A). As expected, FASN^high^ ovarian cancer grows faster and was more aggressive than FASN^low^ ovarian cancer in the subcutaneous model (Figure 2A) or in the peritoneal model (Supplementary Figure S2B). Analysis of immune infiltrates revealed that FASN^high^ ovarian cancer contained low CD3^+^ T cells infiltration (Figure 2B). Additionally, the proportion of exhausted cells (Eomes^+^T-bet^−^) in CD8^+^ populations was higher in FASN^high^ tumors (Figure 2C), suggesting T-cell dysfunction in the tumor context. In contrast, the proportion of effector memory cells (CD44^+^CD62L^−^) in CD8^+^ populations was lower in FASN^high^ tumor than in FASN^low^ tumor (Figure 2D). Tumor-infiltrating CD4^+^ T cells and CD8^+^ T cells displayed the similar phenotype in FASN^high^ OvCa (Supplementary Figures S2C,D). However, FoxP3^+^ regulatory T cells (Tregs) were detected with no difference (data not shown). Consistent with this phenotype, CD3^+^ T cells sorted from FASN^high^ tumors showed defective interleukin (IL)-2 and interferon (IFN)-γ production (Figure 2E, Supplementary Figure S2E). Our data suggested that tumor-intrinsic FASN prevents the early step of T-cell priming against tumor-associated antigens. Recent studies have reported that DCs commonly infiltrate ovarian tumors and promote malignant progression by preventing activation and expansion of tumor-reactive T cells (18, 19). Then, we sought to determine whether FASN might drive tumor growth by inhibiting DCs-dependent anti-tumor immunity. We didn't find the significant difference in the quantification of TIDCs or ascites-infiltrating DCs between FASN^low^ and FASN^high^ OvCa (Figures 2F,G). In consistent with the mouse model, based on TIMER analysis, human TCGA dataset also presented no significant difference between the distributions of DCs and each copy number status of FASN in ovarian cancer (Supplementary Figure S3). However, DCs expressing low levels of co-stimulatory molecules were present in tumor-draining LNs (DLNs) of mice with FASN^high^ tumors (Figures 2H,I). Remarkably, we also found that sorted MHC-II^+^CD11c^+^ DCs from FASN^high^ OvCa DLNs elicited comparable dysfunctional activity (Figure 2J). In contrast, DCs derived from the DLNs of FASN^low^ OvCa did not impair the expansion of CD8^+^ T cells (Figure 2J). Hence, these data indicate that DCs in the microenvironment of FASN^high^ OvCa exhibit marked downregulation of costimulatory markers and dysfunctional activity.

### Lipid Accumulation in Tumor-Infiltrating DCs Derived From the FASN^high^ Ovarian Cancer Accounted for Its Defective Function

**Figure 3:**
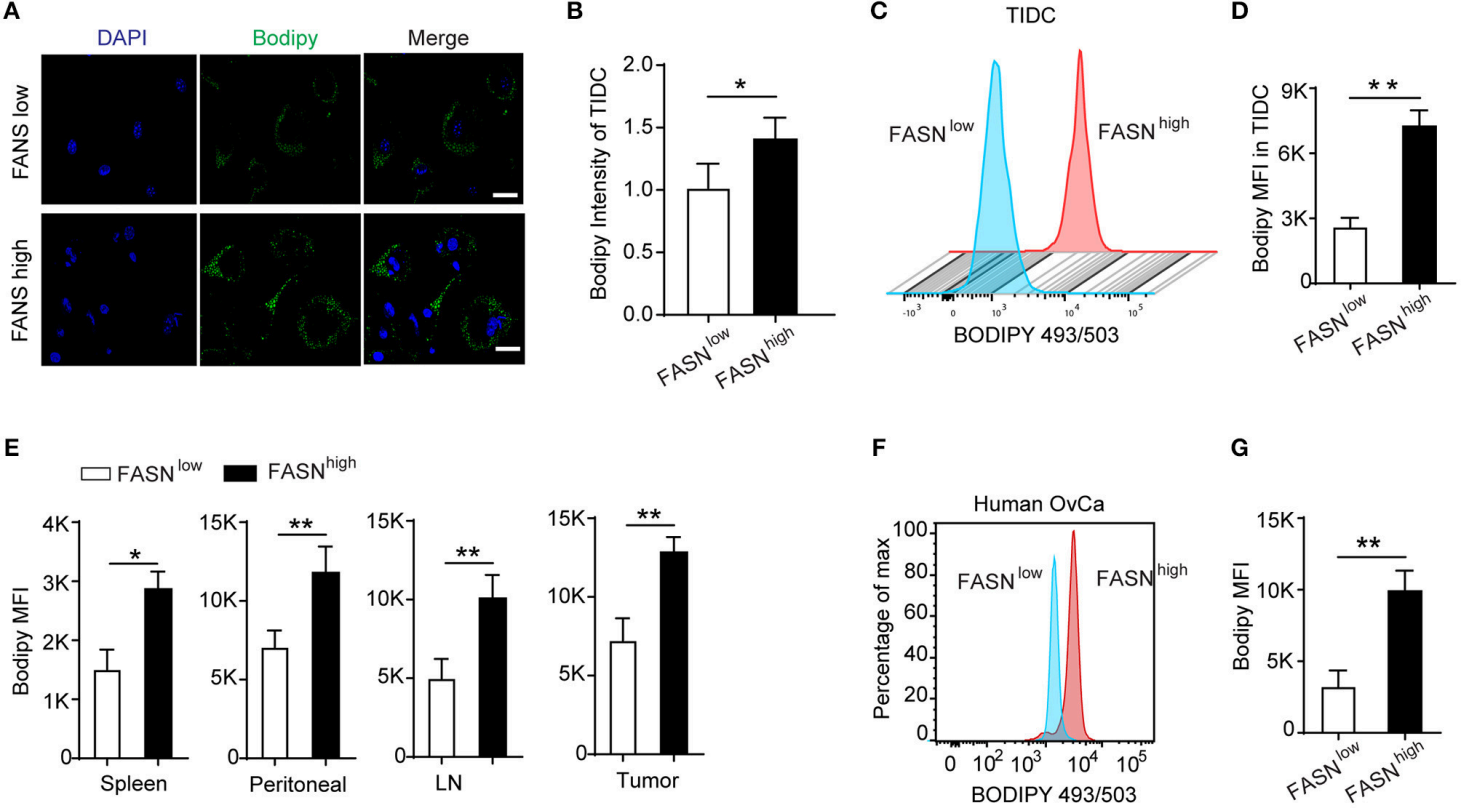
Lipid accumulation in tumor-infiltrating DCs derived from the FASN^high^ ovarian cancer accounted for its defective function. **(A)** Staining of sorted tumor-infiltrating dendritic cells (TIDCs) from FASN^low^ or FASN^high^ ID-8 tumor-bearing mice with BODIPY 493/503. Representative images are shown; scale bar, 20 μm. Green, BODIPY 493/503; blue, DAPI. **(B)** Immunofluorescence intensity of BODIPY 493/503 in TIDCs was measured by Image J and quantification was shown (*n* = 5). **(C)** Flow cytometry analysis of lipid level in TIDC from FASN^low^ or FASN^high^ ID8 tumor-bearing mice. **(D)** Geometric mean fluorescence intensity (MFI) of BODIPY 493/503 in TIDCs was measured. Each group includes five mice. FASN^low^ and FASN^high^ ID8 tumor-bearing mice were evaluated in parallel in each experiment. **(E)** Quantification of lipid level in MHC-II^+^CD11c^+^ DCs from spleen, ascites, lymph node, and tumor in FASN^low^ or FASN^high^ ID-8 tumor-bearing mice. **(F)** Flow cytometry analysis of BODIPY 493/503 staining of TIDCs from an individual with ovarian cancer showed human TIDCs also have high lipid level. **(G)** Cumulative results of lipid levels in TIDCs from individuals with OvCa. Data = mean ± SEM; representative of at least 3 independent experiments; ^*^P < 0.05; ^**^P < 0.01.

Recent evidence has reported that upon infiltrating the tumor microenvironment, myeloid cells including DCs increase the uptake of fatty acids that eventually leads to the upregulation of their immunosuppressive function (26, 27, 35). In an attempt to understand the mechanism of dysfunction by TIDCs in FASN^high^ OvCa, we evaluated the distribution of lipid in TIDCs. After gating the TIDCs (Supplementary Figure S4), the lipophilic fluorescent dye BODIPY 493/503 was used to detect the lipid amount in DCs. Immunofluorescence microscopy of TIDCs from ID8 dissociated tumors 2 weeks after engraftment into mice revealed more BODIPY 493/503 in TIDCs than their control counterparts (Figures 3A,B). Flow cytometry analysis revealed a significantly increased lipid content in TIDCs from FASN^high^ OvCa (Figures 3C,D), confirming the lipid accumulation in TIDCs from FASN^high^ OvCa. We also measured the lipid content in FASN^high^ OvCa as well as several DCs-containing compartments (spleen, lymph node, and peritoneal cavity). In tumor-bearing OvCa mice, DCs isolated from FASN^high^ OvCa displayed higher levels of lipids than DCs from FASN^low^ OvCa (Figure 3E). Given these observations in mice, we wondered whether human TIDCs derived from FASN^high^ OvCa similarly have higher lipid accumulation. Indeed, we found that consistent with what was observed in mice, TIDCs have elevated lipid content in OvCa patients with higher FASN expression (Figures 3F,G).

### The FASN-High Ovarian Tumor Microenvironment Is Rich in Lipids

**Figure 4:**
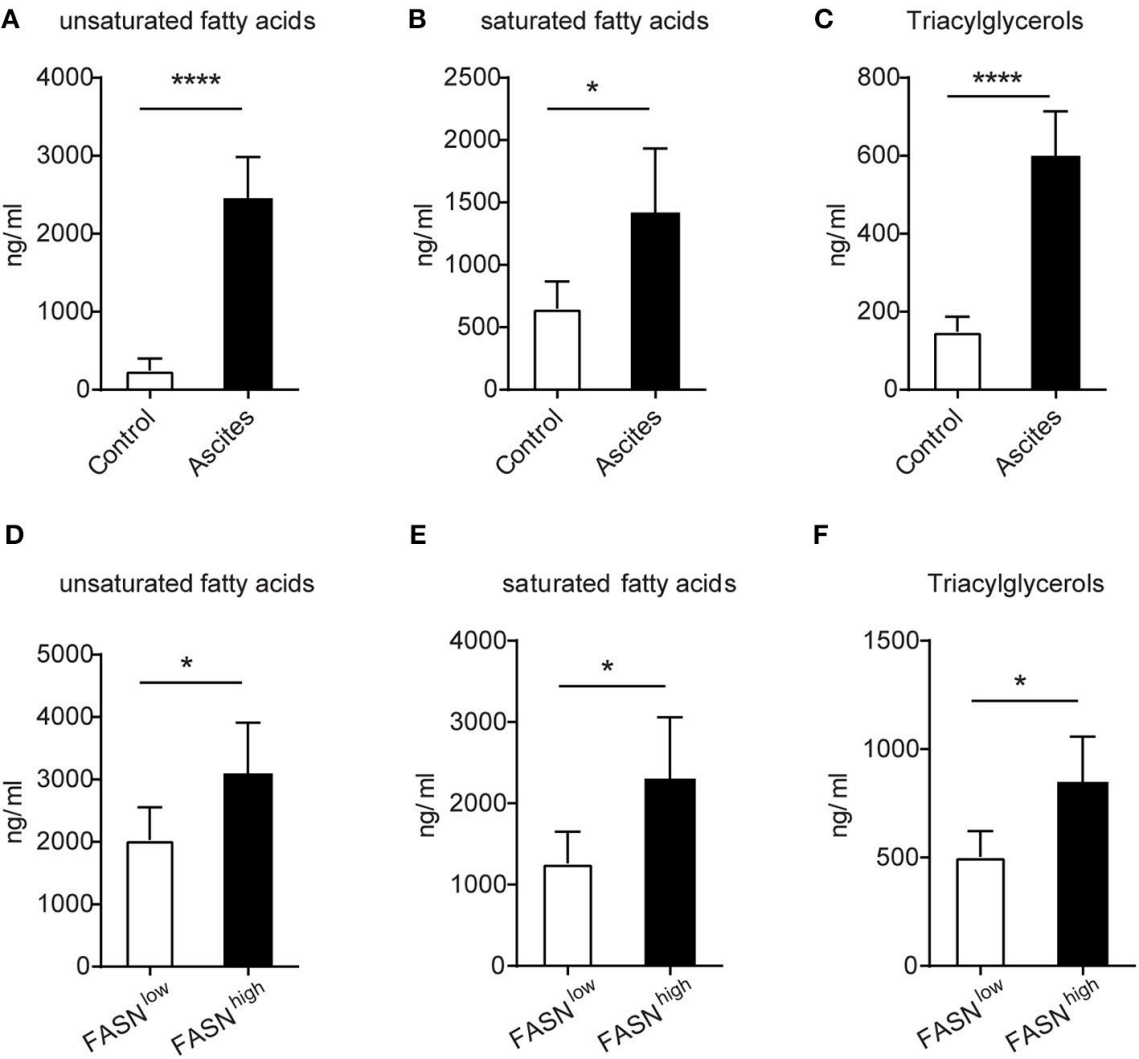
The enrichment of lipids in the FASN-high ovarian tumor microenvironment. **(A–C)** The supernatants of malignant ascites were collected from ID-8 tumor-bearing mice after 3 weeks. The normal peritoneal fluid was collected from naive C57BL/6J mice. The amount of fatty acids and triacylglycerols were quantitatively analyzed via LC-MS. Depicted are total unsaturated fatty acids **(A)**, saturated fatty acids **(B)**, and triacylglycerols **(C)**. **(D–F)** C57BL/6J mice were intraperitoneally injected with 5 × 10^5^ FASN^low^ or FASN^high^ ID8 cells, and supernatants of malignant ascites were collected after 3 weeks. Fatty acids and triacylglycerols were extracted and quantitatively analyzed by LC-MS. Depicted are total unsaturated fatty acids **(D)**, saturated fatty acids **(E)**, and triacylglycerols **(F)**. Data = mean ± SEM; representative of 2 independent experiments; ^*^P < 0.05; ^****^P < 0.0001.

**Figure 5:**
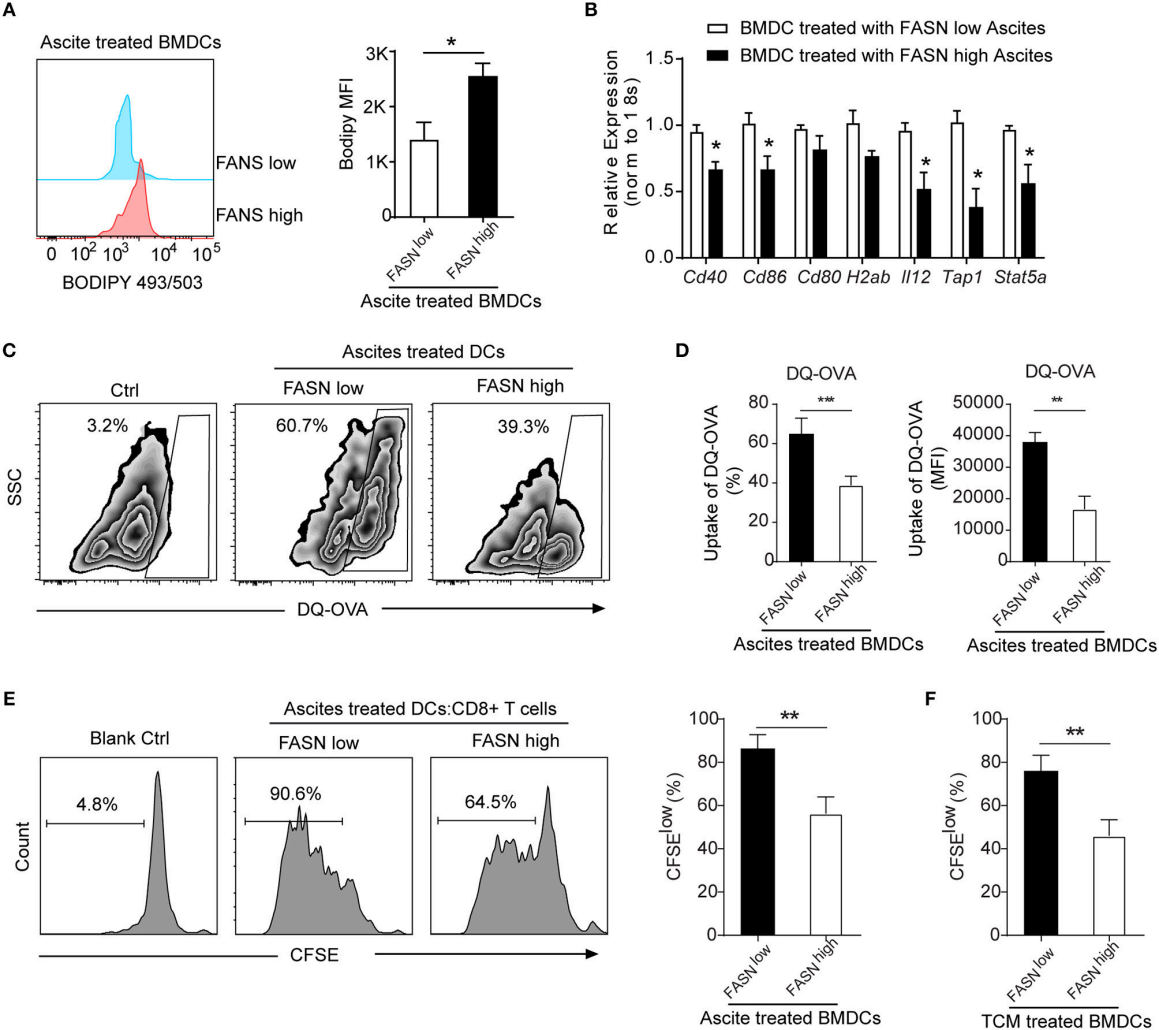
Educated DCs by ascites exhibited defective antigen presentation and impaired priming of T cell activation. **(A)** Flow cytometric staining with Bodipy 493/503 in BMDCs cultured in the presence of 20% ascites harvested from FASN^low^ or FASN^high^ ID-8 tumor-bearing mice for 24 h. MFI average is depicted. Means ± s.d. are shown. **(B)** mRNA expression of indicated DCs associated genes in BMDCs cultured in the presence of 20% ascites harvested from FASN^low^ or FASN^high^ ID-8 tumor-bearing mice. The results were normalized to the level of expression of 18sRNA. **(C,D)** Uptake of DQ-OVA by BMDCs in the presence of 20% ascites harvested from FASN^low^ or FASN^high^ ID-8 tumor-bearing mice. Quantification of uptaking DQ-OVA was shown. Means ± s.d. of three mice per group are shown. **(E, F)** *In vitro* immunostimulatory activity of BMDCs treated with ascites **(E)** or TCM **(F)** harvested from FASN^low^ or FASN^high^ ID8 tumor-bearing mice. Representative histograms of CD8^+^ T cell proliferation at CD8^+^ to BMDCs ratio 10:1 (left panel) and quantification of CD8^+^ T cell proliferation using CFSE dilution (right panel) (*n* = 3). Data = mean ± SEM; representative of at least 3 independent experiments; ns, no significance; ^*^P < 0.05; ^**^P < 0.01; ^***^P < 0.001.

Due to the high lipids in TIDCs from FASN^high^ OvCa, we wondered if there was an increase of lipids in the tumor microenvironment of FASN^high^ OvCa. The lipidomic analysis was analyzed in ascites of ID8 bearing mice collected 3 weeks after tumor implantation and normal peritoneal fluid from control mice. Analysis of lipid content by ESI-MS showed significantly higher concentrations of unsaturated fatty acids, saturated fatty acids (Figures 4A,B) and triacylglycerols (Figure 4C) in ascites of ID8 ovarian cancer-bearing mice. As expected, compared to FASN^low^ OvCa bearing mice, there were significantly higher concentrations of unsaturated fatty acids, saturated fatty acids, and triacylglycerols in ascites of ID8 ovarian cancer-bearing mice with higher FASN expression (Figures 4D–F). To directly determine the source of the high lipid level, we analyzed the tumor cell conditioned medium (TCM). Analysis of lipid content also revealed that tumor-conditioned medium from ID8 cell with higher FASN expression had a higher level of triacylglycerol and fatty acids (data not shown). To test whether these lipids in tumor microenvironment would promote the conversion of DCs into metabolically inactive DCs, we generated DCs *in vitro* from bone marrow by using granulocyte-macrophage colony–stimulating factor and interleukin-4. *In vitro*–generated BMDCs were incubated with ascites derived from FASN^high^ and FASN^low^ OvCa. The result showed more than two-fold increase in the lipid level of BMDCs cultured with ascites from FASN^high^ OvCa than FASN^low^ OvCa (Figure 5A). We observed a similar effect with TCM from other ovarian tumors (data not shown). Analysis of DC-associated gene signature by RT-PCR also revealed that DCs generated in the presence of ascites with FASN^high^ OvCa had decreased expression of costimulatory genes than FASN^low^ OvCa cancer (Figure 5B). Taken together, our data suggested that FASN upregulated lipid generation in the tumor microenvironment to induce the lipid accumulation of DCs.

### FASN-High OvCa Ascites Induce DCs to Dampen Antigen Presentation and T Cell Activation

To investigate whether lipid accumulation in DCs has functional consequences, DCs was generated *in vitro* from bone marrow and educated with ascites from FASN^high^ or FASN^low^ ID8 tumor-bearing mice. To assess the ability of DCs to process antigen, a self-quenching conjugate of DQ ovalbumin (DQ-OVA) was used to show fluorescence upon proteolytic degradation, and the percentage and fluorescent intensity of uptake DQ-OVA were analyzed by flow cytometry. BMDCs educated by ascites from FASN^high^ OvCa tumor-bearing mice had a substantially lower DQ-OVA than from FASN^low^ OvCa mice (Figures 5C,D). We subsequently investigated whether lipid accumulation in DCs stimulates allogeneic T cells. Educated DCs with ascites was co-cultured with CD8^+^ T cell at 1:10 ratio with appropriate stimulation. DCs educated by ascites from ID8 FASN^low^ tumor-bearing mice had certain promoting effects on allogeneic T cell proliferation, whereas ascites derived from FASN^high^ OvCa inhibited the ability of DCs generated from wild-type BMPs to stimulate allogeneic T cells (Figure 5E). Similarly, DCs educated by TCM from FASN^high^ ID8 cells also had a substantially lower stimulatory effect for T cells proliferation than by TCM from FASN^low^ cells (Figure 5F).

### Inhibition of FASN Suppress OvCa Progression by Inducing Anti-Tumor Immunity

**Figure 6:**
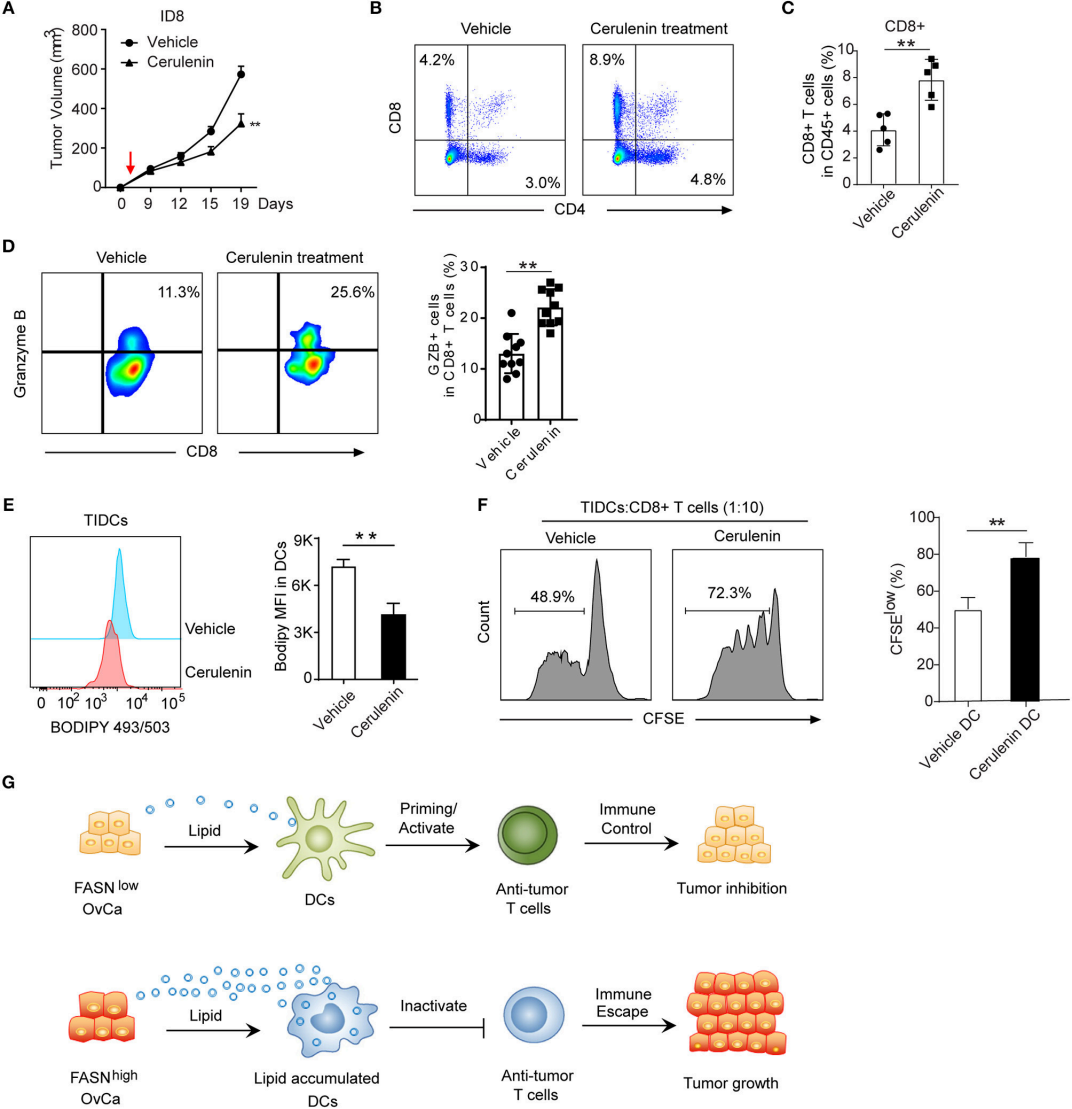
Inhibition of FASN suppress OvCa progression by inducing an anti-tumor immune response. **(A)** Mean tumor volume of the subcutaneous ID8 tumor with the treatment of vehicle or cerulenin (*n* = 10). The red arrow indicated the start of cerulenin treatment. **(B)** Representative flow cytometric analysis of CD8^+^ and CD4^+^ T cells populations in ID8 tumors with or without the treatment of cerulenin at 15 days post-implantation. **(C)** Quantification of CD8^+^ T cells population in ID8 tumors with or without the treatment of cerulenin at 15 days post-implantation (*n* = 5). **(D)** Representative flow cytometric analysis and quantification of Granzyme B expression on CD8^+^ T cells population in ID8 tumor with or without cerulenin treatment at 15 days post-implantation. (*n* = 10). **(E)** Representative flow cytometric analysis and quantification of lipid level in TIDCs sorted from the ID8 tumor with or without cerulenin treatment (*n* = 5). **(F)** *In vitro* immunostimulatory activity of TIDCs sorted from the ID8 tumor with or without the treatment of cerulenin (*n* = 5). Representative histograms and quantification of CD8^+^ T cell proliferation using CFSE dilution (right panel) were shown (*n* = 3). Data presented as mean ± SEM; ^**^P < 0.01. **(G)** Summary of crosstalk between FASN^low^ or FASN^high^ tumor cells and DCs-mediated adaptive immune response as described in the text.

To explore how FASN inhibition affects anti-tumor immune response *in vivo*, we analyzed tumor-infiltrating immune cells in ID8 mice treated with FASN inhibitor cerulenin, a small molecule antibiotic. Indeed, cerulenin treatment led to ID8 tumor growth regression in subcutaneous or intraperitoneal immunocompetent models (Figure 6A, Supplementary Figures S5A,B), but not in nude mice (Supplementary Figure S5C). In addition, *in vitro* assay indicated that the effect of cerulenin on DCs' function is minimal (Supplementary Figure S5D). Flow analysis showed significantly higher tumor-infiltrating CD4^+^ T cells and CD8^+^ CTLs in the tumor microenvironment of FASNi- treated ID8 mice (Figures 6B,C). Consistent with increased infiltration of cytotoxic T cell populations, we also observed more IFN-r^+^ and Granzyme B^+^ functional CD8^+^ T cells and less Eomes^+^ exhausted CD8^+^ T cells in FASN inhibitor-treated OvCa tumor (Figure 6D, Supplementary Figures S5E,F). Furthermore, lipid content was significantly decreased in TIDCs derived from FASN inhibitor-treated ID8 mice compared to the untreated group (Figure 6E). To directly address this function of TIDCs, we sorted TIDCs from tumor tissue in ID8 transplanted mice with or without FASN inhibitor treatment. TIDCs isolated from FASN inhibitor-treated ID8 model displayed the enhanced capacity to induce the proliferation of CD8^+^ T cells, compared with their control counterparts (Figure 6F). Taken together, our data show that OvCa-cell-intrinsic FASN activation can result in the exclusion of the host immune response, including lipid accumulation in tumor-infiltrating dendritic cells and subsequently the absence of a T-cell infiltrate and dysfunction within the tumor microenvironment (Figure 6G).

## Discussion

Here we conclude that ovarian cancer cell-intrinsic activation of an oncogenic FASN pathway can result in the exclusion of the host immune response, including the dysfunction of dendritic cells and the absence of a T-cell infiltrate within the tumor microenvironment. We uncover an unexpected role for tumor intrinsic FASN as a driver of DCs malfunction in the tumor microenvironment by lipid accumulation. We find that FASN signaling is a driver of immunosuppression and OvCa progression. The present study extends the investigation of the potential use of a FASN inhibitor for immunotherapy in mice. In this study, we also revealed the effect of cerulenin on growth inhibition of the syngeneic murine ID8 OvCa model by inhibiting immunosuppression.

Numerous studies have shown that many human cancer cells have high activities of FASN, and the cytotoxic effects of FASN have been described both *in vitro* and *in vivo* (17, 36, 37). However, the role of upregulated FASN in cancer cells and the detailed mechanisms of tumor cells killing by an inhibitor of FASN are still not fully understood. In this study, we have demonstrated that FASN was upregulated in human ovarian cancer and associated with the immunosuppressive microenvironment. FASN signaling has been confirmed as a regulator of multiple signaling pathways during tumor progression, including cell-cell adhesion, migration, proliferation and chemokine transcription (38–40). As such, FASN has been shown to be important in several cancer types, including ovarian cancer (14, 40). However, the role of FASN signaling in driving suppressive tumor microenvironment is less well understood. Recent reports have highlighted the importance role of lipids on the function of immunosuppressive myeloid cells including M2 macrophages, dendritic cells, and MDSC in inflammatory conditions and cancer (26, 27, 35, 41). In recent years, accumulation of lipids was implicated in the defective cross-presentation by tumor-associated DCs (42–44). This suggested that factors in tumor microenvironment could mediate the immunometabolic induction of DCs. Here, we identified that OvCa cancer-intrinsic FASN might initiate this process. OvCa-derived FASN produces abundance lipid that facilitates the uptake of lipids abundant in the tumor microenvironment, including free fatty acids and the triacylglycerol-carrying lipoproteins VLDL and LDL.

A substantial proportion of DCs in tumor-bearing hosts have an increased amount of lipids, specifically triglycerides. In this study we have tried to determine the mechanism of lipid accumulation in DCs and whether it has any functional consequences for these cells. Accumulation of lipids might be due to increased synthesis of fatty acids in FASN^high^ OvCa cell and then result from increased lipid uptake from tumor microenvironment. Importantly, human cancer-associated DCs also express lipid transporters and therefore BMDCs cultured in FASN^high^ OvCa conditioned media to develop into highly defective DCs. The uptake and accumulation of these lipids support the activation of immunodefective DCs. Several reports have demonstrated that dendritic cells in ovarian cancer correlated with clinical outcome in patients with ovarian cancer (45). In addition, ovarian cancer progresses involving the recruitment of immunostimulatory DCs that induce measurable T cell-mediated anti-tumor immunity (19). Our findings are also significant as we show an abundance of lipids in the tumor microenvironment of FASN^high^ OvCa cancer that can be acquired by DCs. This observation supports previous reports that show increased levels of triglycerides, HDL-cholesterol in the circulation of ovarian cancer patients (46, 47). Moreover, the T-cell-inflamed tumor microenvironment phenotype appears to be predictive of clinical response to immune-based therapies (48). Immune escape among this subset appears to be a consequence of dominant effects of negative regulatory pathways such as PD-1, arguing that the clinical activity of anti-PD-1 is tipping the balance in favor of an ongoing immune response. By inference, tumor-intrinsic FASN activation may represent one mechanism of primary resistance to these therapies.

Taken together, our findings suggest a critical role of lipid uptake and accumulation in the metabolic and functional reprogramming in DCs of ovarian cancer. The regulation of these processes by tumor-derived FASN-dependent mechanisms provide an opportunity to simultaneously target the multiple immunosuppressive pathways harnessed by tumor-associated DCs. These findings indicate that tumor cell-intrinsic FASN signaling may be a driver of immune escape in ovarian cancer, and thus may be a target for combination with immunotherapy in ovarian cancer.

## Author Contributions

XW and LJ supervised the whole project, designed the experiments, analyzed data, and wrote the manuscript. LJ and XF performed the most experiments, analyzed data, and prepared the figures. HW and DL contributed to some experiments and provided the technical support. All authors read and approved the final manuscript.

### Conflict of Interest Statement

The authors declare that the research was conducted in the absence of any commercial or financial relationships that could be construed as a potential conflict of interest.

## Abbreviations

OvCa: ovarian cancer
TIDCs: tumor-infiltrating dendritic cells
DCs: dendritic cells
FASN: fatty acid synthase
TME: tumor microenvironment
TIMER: tumor immune estimation resource
FMOs: fluorescence minus one controls
MDSC: myeloid-derived suppressor cell
TCM: tumor-conditioned medium
TES: tumor explant supernatant
Tregs: regulatory T cells.
