# Supplementary material for: Ovarian Cancer-Intrinsic Fatty Acid Synthase Prevents Anti-tumor Immunity by Disrupting Tumor-Infiltrating Dendritic Cells
Source: Front Immunol. 2018 Dec 14;9:2927. doi: 10.3389/fimmu.2018.02927 (PMC6302125; doi:10.3389/fimmu.2018.02927)
Supplement: Supplementary file 1 [file Data_Sheet_1.docx]

Supplementary Material

**Ovarian Cancer-Intrinsic Fatty Acid Synthase Prevents Anti-Tumor Immunity by Disrupting Tumor-infiltrating Dendritic Cells**

Li Jiang^1^*^,†^, Xuhong Fang^1†^, Hong Wang^1^, Diyou Li^1^, Xipeng Wang^1^*

^1^Department of Gynecology and Obstetrics, Xinhua Hospital, Shanghai Jiao Tong University School of Medicine, 1665 Kongjiang Road, Shanghai 200092, China.

^†^ These authors have contributed equally to this work

* Correspondence:

Li Jiang, E-mail: jiangli@xinhuamed.com.cn

Xipeng Wang, E-mail: xipengwang@hotmail.com

# Supplementary Figures and Tables

## Supplementary Table

**Supplementary Table S1. Antibody list for Flow cytometry.**

| **Name** | **Host** | **Clone** | **Company** | **Dilution** | |
| --- | --- | --- | --- | --- | --- |
| CD45-APC/Cy7 | Mouse | 30-F11 | Biolegend | 1:100(FC) |  |
| CD11b-PE/Cy7 | Mouse/Human | M1/70 | Biolegend | 1:50(FC) |  |
| Ly6G- PerCP/Cy5.5 | Mouse | IA8 | eBioscience | 1:50(FC) |  |
| F4/80-PE | Mouse | BM8 | eBioscience | 1:50(FC) |  |
| CD11c-PE | Mouse | N418 | Biolegend | 1:100(FC) |  |
| CD44-PE/Cy7 | Mouse | IM7 | Biolegend | 1:100(FC) |  |
| CD62L-FITC | Mouse | MEL-14 | Biolegend | 1:100(FC) |  |
| T-bet-FITC | Mouse | 4B10 | Biolegend | 1:100(FC) |  |
| Eomes-PE | Mouse | WD1928 | eBioscience | 1:100(FC) |  |
| Granzyme B | Mouse | GB11 | Biolegend | 1:100(FC) |  |
| I-A/I-E-FITC | Mouse | M5/114.15.2 | Biolegend | 1:100(FC) |  |
| CD40-APC | Mouse | 3/23 | Biolegend | 1:100(FC) |  |
| CD86-PerCP/Cyanine5.5 | Mouse | GL-1 | Biolegend | 1:100(FC) |  |
| CD4-FTIC | Mouse | GK1.5 | Biolegend | 1:100(FC) |  |
| CD8- PerCP/Cyanine5.5  IFN-r-APC  CD3 PE-Cy7  CD103 PerCP-eFluor 710  FoxP3-APC | Mouse  Mouse  Mouse  Mouse  Mouse | 53-6.7  XMG1.2  145-2C11  2E7  3G3 | Biolegend  eBioscience  eBioscience  eBioscience  eBioscience | 1:100(FC)  1:100(FC)  1:100(FC)  1:100(FC)  1:100(FC) |  |

## Supplementary Figures

**
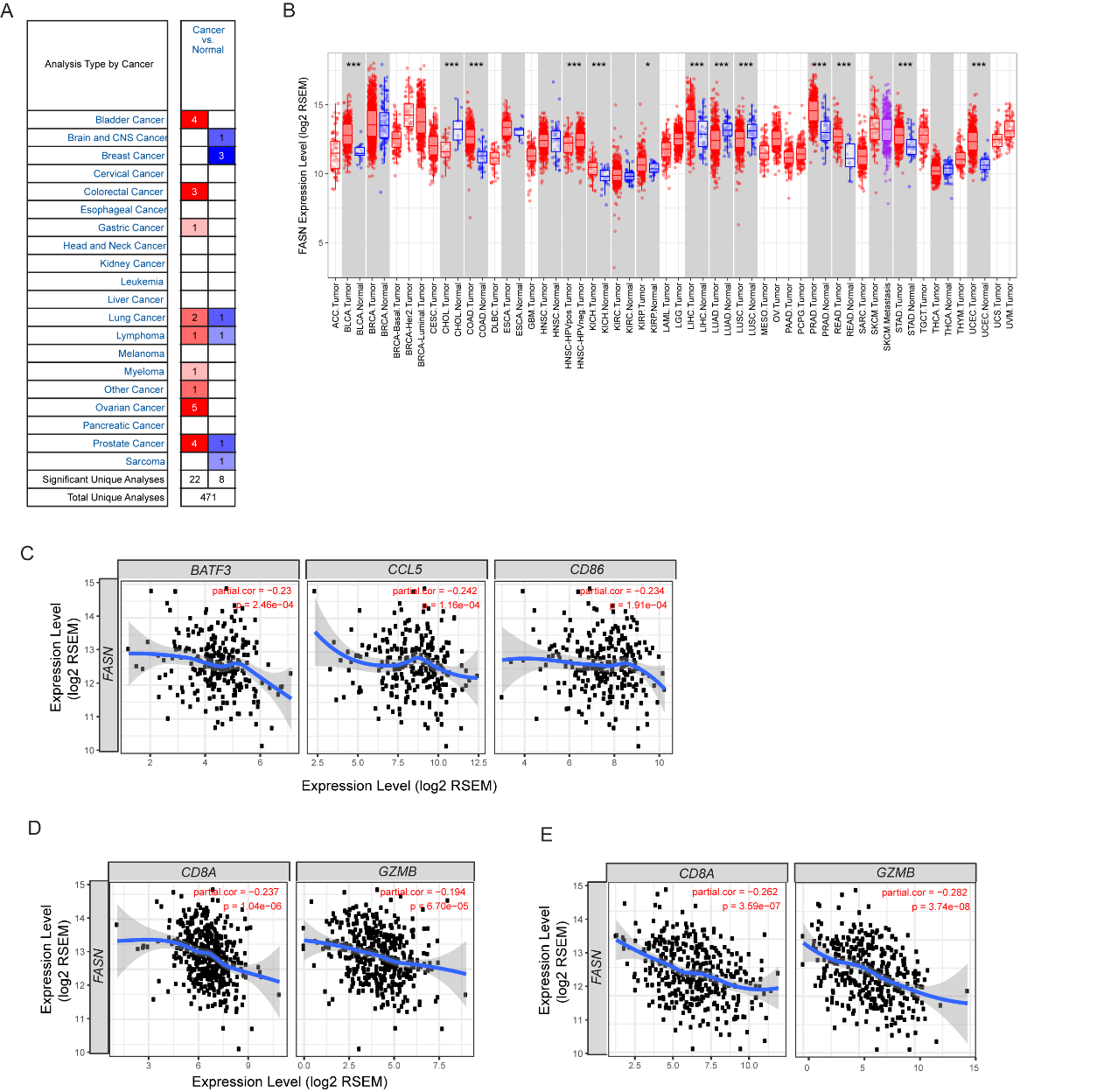
**

**Supplementary Figure S1. FASN was overexpressed in cancer progression and negatively correlated with anti-tumor immune response genes.** **(A),** Heatmap showed that the up-regulation of FASN was found in 9 of 20 cancer types through Oncomine data mining analysis. **(B),** Box plots with the statistical significance of the differential expression evaluated the expression of FASN between tumor and adjacent normal tissues across all TCGA tumors using Wilcoxon test. **(C),** The correlation of FASN mRNA expression with gene signature of CD11c^+^ DC cells (*BATF2, CCL5,* and *CD86*) in human ovarian cancer based on TIMER analysis from TCGA datasets. The scatterplots showed the purity-corrected partial Spearman’s correlation and statistical significance. **(D, E),** The correlation of FASN mRNA expression with cytotoxic T cells gene (*CD8A* and *GZMB*) in human prostate cancer (D) and bladder cancer (E) based on TIMER analysis from TCGA datasets. The scatterplots showed the purity-corrected partial Spearman’s correlation and statistical significance.

**
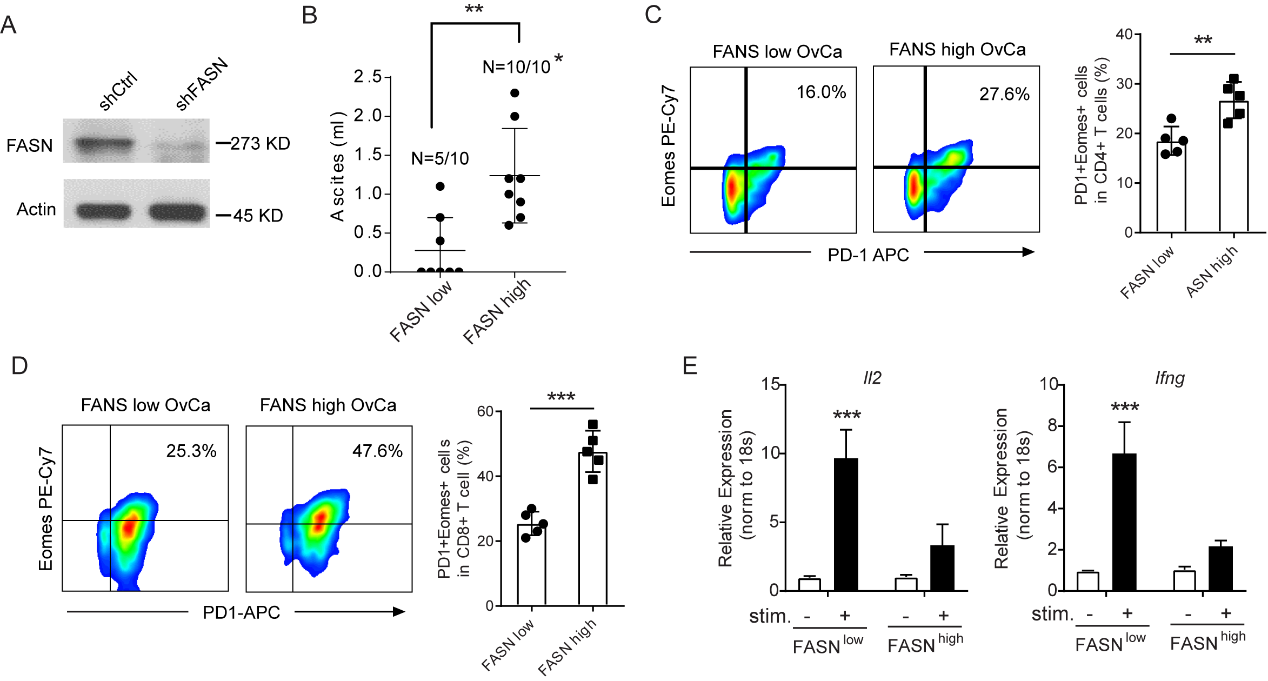
**

**Supplementary Figure S2. Tumor-intrinsic FASN impaired anti-tumor immune response. (A),** Validation of FASN expression in shCtrl-ID8 cell and shFASN-ID8 cell by western blot. **(B),** Scatterplots representing the incidence and volume of ascitic fluid measured in tumor-bearing mice at the end of 8 weeks after shCtrl-ID8 or shFASN-ID8 injection. Values represent mean ± SEM. **P* < 0.05, comparing the incidence by χ^2^ test. ***P*<0.01, compared with the ascites volume in shCtrl-ID8 mice. **(C, D)** Representative flow cytometry analysis and quantification of PD-1^+^Eomes^+^ exhausted CD4^+^ T cells (C) and PD-1^+^Eomes^+^ exhausted CD8^+^ T cells (D) from mice transplanted with shCtrl-ID8 or shFASN-ID8 tumor. **(E).** Il2 and Ifng transcripts present in sorted CD3^+^ T cells from FASN^low^ or FASN^high^-ID8 tumors with or without anti-CD3/CD28 stimulation (n=5). Data=mean ± SEM; representative of at least 5 independent experiments; *, *P* < 0.05; **, *P* < 0.01; ***, *P* < 0.001.

**
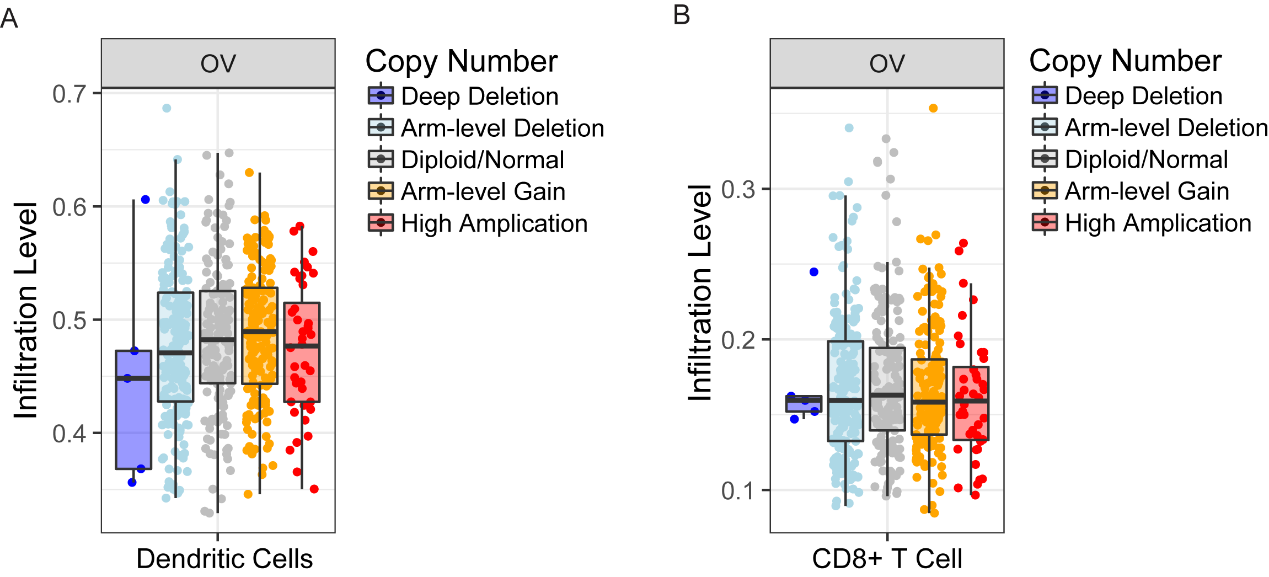
**

**Supplementary Figure S3. The abundance of tumor-infiltrating immune cells among ovarian cancer with somatic copy number alteration (SCNA) of FASN. (A)**, Box plot is presented to show the distributions of tumor-infiltrating dendritic cells for each copy number status of FASN in human ovarian cancer. **(B)**, Box plot is presented to show the distributions of tumor-infiltrating CD8^+^ T cells for each copy number status of FASN in human ovarian cancer. SCNAs are defined by GISTIC 2.0, including deep deletion, shallow deletion, diploid/normal, low-level gain, and high amplification.

**
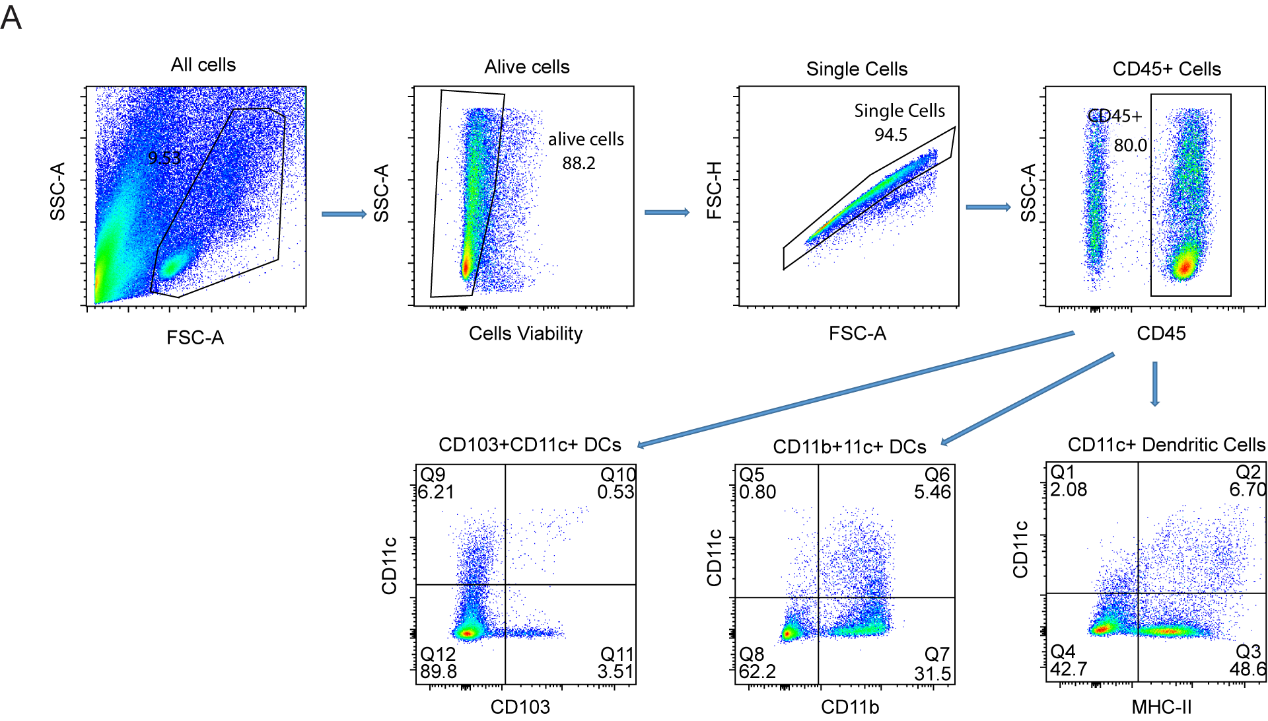
**

**Supplementary Figure S4. The flow cytometry gating strategies for analyzing tumor-infiltrating dendritic cells (TIDCs).** (**A**), initial gating was on immune cells as approximated by scattering, then dead cells and doublets were eliminated. Next, CD45^+^ cells were selected, CD11c^+^MHC-II^+^ double positive cells were gated for total dendritic cells (DCs), and then analyzed the CD11b^+^CD11c^+^ double positive cells as conventional DCs-2 (cDC-2), CD103^+^CD11c^+^ double positive cells as conventional DCs-1 (cDC-1).

**
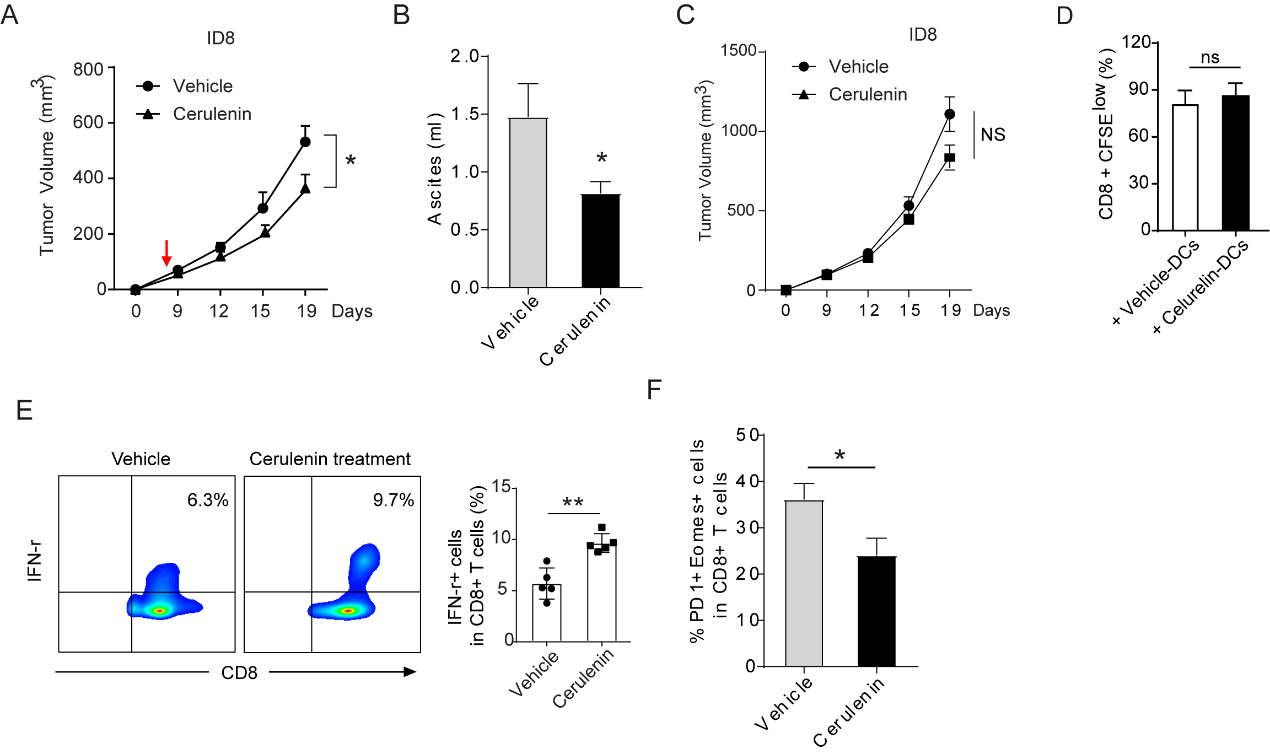
**

**Supplementary Figure S5. Inhibition of FASN by cerulenin suppress OvCa progression by inducing an anti-tumor immune response.** (**A**), the tumor growth of the ID8 tumor with the treatment of vehicle or cerulenin at day 7, 10, 13. (n = 10). The red arrow indicated the start of cerulenin treatment. (**B**), the volume of ascitic fluid measured in tumor-bearing mice at the end of 8 weeks after ID8 injection with or without cerulenin treatment. (**C**), Tumor growth in nude mice after the inoculation of ID-8 cell treated with vehicle control or cerulenin. (**D**), CD11c^+^ DC cells treated with or without cerulenin 24h, and then cocultured with CD8^+^ T cell at ratio 1:10 (DC: CD8^+^ T cells). Quantification of CD8^+^ T cell proliferation using CFSE dilution was shown (n = 3). (**E**), Representative flow cytometric analysis and quantification of IFN-r expression on CD8^+^ T cells population in ID8 tumor with or without the treatment of cerulenin at 14 days post implantation (n = 5). (**F**), Quantification of PD-1^+^Eomes^+^ exhausted CD8^+^ T cells from mice transplanted with ID8 tumor with or without cerulenin treatment at 14 days post implantation (n = 5). Data presented as mean ± SEM. NS, no significance; *, *P* < 0.05; **, *P* < 0.01.
